# First metagenomic analysis of age-associated changes in the gut microbiome among healthy Saudi adults: SAMS pilot study

**DOI:** 10.3389/fragi.2026.1733638

**Published:** 2026-03-11

**Authors:** Roua Almatrafi, Abdulrahman Alasiri, Ghaida Almuneef, Amal A. Al-Hazzani, Majed F. Alghoribi, Maymounah Hakami, Assad M. Arafah, Raniah S. Alotibi, Shatha Alrabiah, Nasser Alqurainy, Reham Ajina, Marwh G. Aldriwesh

**Affiliations:** 1 Department of Botany and Microbiology, College of Science, King Saud University, Riyadh, Saudi Arabia; 2 Infectious Disease Research Department, King Abdullah International Medical Research Center, Riyadh, Saudi Arabia; 3 Department of Artificial Intelligence and Data Management, Bioinformatics Division, King Abdullah International Medical Research Center, King Saud bin Abdulaziz University for Health Sciences, Riyadh, Saudi Arabia; 4 Ministry of National Guard-Health Affairs, Riyadh, Saudi Arabia; 5 Department of Basic Science, College of Science and Health Professions, King Saud bin Abdulaziz University for Health Sciences, Riyadh, Saudi Arabia; 6 King Abdullah International Medical Research Center, Riyadh, Saudi Arabia; 7 Department of Family Medicine, King Abdulaziz Medical City, Ministry of National Guard Health Affairs, Riyadh, Saudi Arabia; 8 College of Medicine, King Saud Bin Abdulaziz University for Health Sciences, Riyadh, Saudi Arabia; 9 Department of Clinical Laboratory Sciences, College of Applied Medical Sciences, King Saud Bin Abdulaziz University for Health Sciences, Riyadh, Saudi Arabia; 10 Department of Clinical Nutrition, College of Applied Medical Sciences, King Saud Bin Abdulaziz University for Health Sciences, Riyadh, Saudi Arabia

**Keywords:** age-associated changes, gut microbiome, healthy adults, metagenomics, Saudi Arabia

## Abstract

**Introduction:**

The gut microbiome undergoes dynamic changes with aging across diverse healthy populations. However, data from Saudi Arabia remain limited. This pilot study investigated age-related variations in the gut microbiome among healthy Saudi adults to characterize region-specific microbial signatures and identify taxa potentially associated with aging in a healthy population.

**Methods:**

We established the Saudi Aging and Microbiome Study (SAMS) to investigate age-related changes in fecal microbiome of Saudi adults. In this pilot phase, 145 healthy participants aged 19–69 years were enrolled. Shotgun metagenomic sequencing was performed to profile fecal microbiome at the species level. Microbial diversity and taxonomic composition were compared across five age groups. Spearman and confounder-adjusted partial Spearman correlation were applied to identify taxa significantly associated with chronological age.

**Results:**

We analyzed fecal microbiome of 145 healthy adults distributed among five age groups: G1 (19–29 years, *n* = 33; 22.7%), G2 (30–39 years, *n* = 30; 20.7%), G3 (40–49 years, *n* = 27; 18.6%), G4 (50–59 years, *n* = 31; 21.4%), and G5 (60–69 years, *n* = 24; 16.6%). Of these, 75 (51.7%) were male, and 70 (48.3%) were female. Alpha diversity increased from young to older adulthood for observed richness and Shannon indexes (all *q* < 0.05). Beta diversity also varied significantly with age (PERMANOVA *R* ^*2*^ = 0.13, *q* = 0.023), indicating distinct microbial community structures in healthy older adults. At the phylum level, *Firmicutes* significantly increased with age (FC = 1.35; *q* = 0.026), whereas *Bacteroidota* decreased (FC = 0.59; *q* = 0.01). Consistent with these trends, *Blautia obeum* showed positive correlations, while *Bacteroides thetaiotaomicron* and *Phocaeicola vulgatus* showed negative correlations with chronological age.

**Conclusion:**

In healthy Saudi adults, increasing age was associated with higher microbial diversity and compositional shifts at phylum and species levels. These age-associated microbial taxa might represent biomarkers of healthy aging and suggest an enhanced community capacity for short-chain fatty acids (SCFAs) production, a hypothesis warranting validation through future functional analyses.

## 1 Introduction

The gut microbiome is a diverse community of microorganisms that resides in the large intestine and plays a crucial role in human health and disease (Sekirov et al., 2010; Thursby and Juge, 2017). The term core gut microbiome commonly refers to the set of microbial taxa found in more than half of individuals within a given population or cohort (Piquer-Esteban et al., 2022; Mancabelli et al., 2024). Bacteria constitute approximately 90% of this community, alongside smaller proportions of archaea, fungi, protists, and viruses (Bäckhed et al., 2005; Lynch and Pedersen, 2016). For human health, a diverse and stable gut microbial ecosystem is essential for maintaining the balance of the core microbiome and supporting overall microbial homeostasis (Lozupone et al., 2012; Rinninella et al., 2019).

Accumulating evidence demonstrates that the gut microbiome undergoes substantial age-related restructuring that influences immune function, metabolic regulation, and overall lifespan, thereby positioning the gut microbiome as a key modulator of human aging (Li and Roy, 2023). Gut microbiome composition varies across the lifespan and is influenced by environmental factors, dietary patterns, and host-microbe interactions (Kim and Benayoun, 2020; Bradley and Haran, 2024). In healthy aging, the gut microbiome is characterized by high microbial diversity and enrichment of short-chain fatty acid (SCFA)-producing taxa, which have been associated with reduced systemic inflammation, improved physical and cognitive function, and lower metabolic risk (Cryan and Dinan, 2012; Zhang et al., 2013; Vogt et al., 2017; Langsetmo et al., 2019; Ghosh et al., 2022). However, age-related dysbiosis, characterized by a reduction in beneficial microbes and an increase in potentially pathogenic taxa, has been recognized as a hallmark of aging and is associated with elevated pro-inflammatory signaling (Wu et al., 2021; López-Otín et al., 2023).

Previous studies from diverse populations, including cohorts from Italy, China, the United States of America, South Korea, and Ireland, have reported age-related changes in the microbial community composition (Jeffery et al., 2016; Kim et al., 2019; Wu et al., 2020; Wilmanski et al., 2021; Ai et al., 2024). In many cohorts, healthy adults showed greater relative abundance of SCFA-producing genera such as *Faecalibacterium*, *Roseburia*, *Butyricicoccus*, and *Blautia*, while unhealthy aging is often associated with decreased levels of *Bifidobacterium* and increased representation of *Proteobacteria* and other enterobacteria, patterns frequently linked to low-grade inflammation and age-related diseases (Hopkins et al., 2001; Biagi et al., 2010; Lemon et al., 2012; Wu et al., 2019; Hou et al., 2022; Li et al., 2022; Pang et al., 2023).

Despite substantial global progress in characterizing age-related changes in the gut microbiome, there remains a considerable lack of data from the Gulf region, particularly Saudi Arabia. This knowledge gap is critical, as the Saudi population is experiencing a demographic transition marked by a growing proportion of older adults and a concomitant rise in age-associated chronic diseases (Al-Amoud et al., 2023). Many of these diseases (such as diabetes, cardiovascular diseases, and neurodegenerative disorders) are associated with gut microbiome dysbiosis (Buford, 2017). Addressing these health challenges aligns closely with the goals of Saudi Vision 2030, which seeks to enhance the quality of life and reduce the burden of chronic diseases that significantly impact public health and the national economy. Establishing baseline gut microbiome data in healthy Saudi adults is therefore essential for identifying both shared and region-specific microbial features associated with age-related changes, thereby contributing to the national and global efforts to promote better health and quality of life.

Most studies of the human gut microbiome and age-related changes have relied on 16S ribosomal RNA (16S rRNA) gene sequencing, which provides genus-level taxonomic resolution but is limited in its ability to identify species-level differences (Badal et al., 2020). In contrast, shotgun metagenomic sequencing enables high-resolution, comprehensive profiling by capturing all microbial DNA within a sample (Ranjan et al., 2016; Bastiaanssen et al., 2019; Li and Lu, 2025). The present pilot study provides the first shotgun metagenomic characterization of the fecal microbiome in healthy Saudi adults among different age groups. Specifically, we aim to (1) profile the diversity patterns across healthy adults, (2) identify the Saudi-specific core healthy adult fecal microbiome, and (3) determine age-associated taxonomic shifts. These analyses offer novel insights into the fecal microbiome and age-related changes in the healthy Saudi population emphasizing both conserved and regionally distinct microbial features with potential implications for public health and the prevention of age-related diseases.

## 2 Methods

The current study was performed at King Abdulaziz Medical City (KAMC), King Abdullah International Medical Research Center (KAIMRC), and King Saud bin Abdulaziz University for Health Sciences (KSAU-HS), which collectively operate under the Ministry of National Guard-Health Affairs (MNGHA) in Riyadh, Saudi Arabia.

### 2.1 Saudi aging and microbiome study (SAMS): cohort design and pilot phase

**FIGURE 1:**
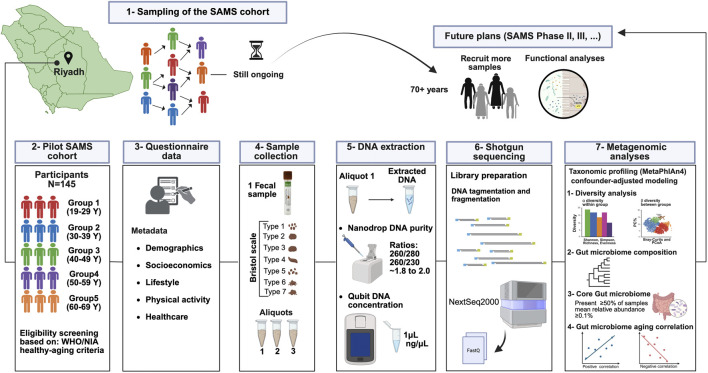
Overview of the Saudi Aging and Microbiome Study (SAMS) workflow. The diagram summarizes participant recruitment via convenience and snowball sampling and eligibility screening based on the World Health Organization (WHO) and National Institute on Aging (NIA) healthy-aging criteria, formation of the SAMS pilot cohort, standardized fecal sample collection, DNA extraction and quality control, shotgun metagenomic sequencing with appropriate controls, and downstream bioinformatics and confounder-adjusted statistical analyses including diversity assessment, taxonomic composition profiling, core microbiome definition, and age-associated correlation analyses. Planned future phases include expansion to advanced age groups and integration of functional multi-omics profiling. The figure was created in BioRender. Aldriwesh, M. (2026) https://BioRender.com/9qmfkxh.

The Saudi Aging and Microbiome Study (SAMS) is an ongoing, longitudinal, population-based study that was established in January 2024 to investigate the association between the fecal microbiome and trajectories of healthy aging in Saudi Arabia. The cohort is being developed in phases. The current pilot phase recruited healthy adults aged 19–69 years to characterize the fecal microbiome associated with age-related changes within the Saudi healthy population. In subsequent phases, the SAMS will include Saudi individuals with age-associated diseases to enable comparative analyses and investigate gut microbiome transitions from health to disease. Figure 1 provides an overview of the SAMS study design, analytical workflow, and future research, including planned expansion to advanced age groups and integration of functional profiling.

In this pilot phase, 145 healthy Saudi adults were recruited for fecal microbiome analysis. Participants were considered healthy if they are absence of major chronic disease, exhibited normal physical and cognitive function, and had social communication. For participants aged 60 years and older, health status was further characterized using criteria commonly applied in aging research, including normal cognitive function (assessed by the Mini-Mental State Examination [MMSE]), the ability to perform daily activities independently, and have social communications, in accordance with the criteria defined by the World Health Organization (WHO, 2020) and the National Institute on Aging (NIA, 2022). The MMSE was administered by the research team in English with on-the-spot Arabic translation; scoring followed the original MMSE criteria. Participants with MMSE ≥24/30 were considered cognitively normal and all enrolled participants in this pilot phase met this criterion. Fecal samples were collected between January 2024 and February 2025 at multiple sites in Riyadh, including the Employee Health Clinic at KAMC and various community locations, using convenience and snowball sampling strategies. Metadata were obtained through face-to-face interviews using a structured questionnaire. Exclusion criteria included a history of cancer, diabetes, hypertension, cardiovascular or chronic lung disease, stroke, peptic ulcer disease, chronic gastrointestinal disorders or infections, and neurodegenerative diseases. Further exclusion criteria included the use of antimicrobials, anti-inflammatory medications, proton-pump inhibitors (PPIs), metformin, or laxatives within the prior 3 months of fecal sample collection. To examine the age-related patterns in the gut microbiome, participants were stratified into five age groups, as described by (Odamaki et al., 2016): G1 (19–29 years; n = 33), G2 (30–39 years; n = 30), G3 (40–49 years; n = 27), G4 (50–59 years; n = 31), and G5 (60–69 years; n = 24). The fecal samples were delivered to KAIMRC, where the analysis of microbial DNA libraries and the bioinformatic analyses of raw sequencing data were performed.

### 2.2 Metadata collection

Face-to-face structured interviews captured demographics (age, sex, body mass index [BMI], job status, marital status, family size), lifestyle (physical activity, frequency of social activity, pet ownership, smoking), sleep difficulty, and nail-biting behavior, as well as medication use (yes/no). Family size was categorized as small (<5 members), medium (5 members), and large (>5 members), following the Saudi Data Centre guideline (Saudi Data, 2024). Social activity was defined as gatherings with family/friends and attendance at social events, then categorized according to frequency as low (none or <1/week), medium (1–3/week), or high (≥4/week), as previously described (Wang et al., 2024). Each fecal sample was evaluated using the Bristol Stool Form Scale at the time of sample collection. Supplementary Table S1 provides information related to the medications and supplements used by the study participants.

### 2.3 Fecal sample collection and microbial genomic DNA extraction

Participants self-collected fresh fecal samples using DNA/RNA Shield Fecal collection tubes (Zymo Research, Cat. No. R1101, Irvine, CA, United States), according to standardized instructions. Then, fecal samples were collected by the research team and transported using biohazard containers on dry ice to the Saudi Biobank Centre at KAIMRC (ISO-accredited biobank center), where samples were accessioned, gently homogenized, aliquoted, and stored at −80 °C. Microbial genomic DNA (gDNA) was extracted using the QIAamp PowerFecal Pro DNA Kit (Qiagen, Cat. No. 51804, Germany), according to the manufacturer’s protocol. DNA concentration was measured using a Qubit™ dsDNA HS Assay Kit (Thermo Fisher Scientific Inc., Cat. No. Q32851; Waltham, MA, United States) and stored at −80 °C until library preparation.

### 2.4 Mock communities and negative controls

A ZymoBIOMICS mock microbial community (Zymo Research, Cat. No. D6300, Irvine, CA, United States) and nuclease-free water (Thermo Fisher Scientific Inc., Cat. No. AM9937; Waltham, MA, United States) were processed as positive and negative controls, respectively, through DNA extraction, library preparation, sequencing, and bioinformatics analyses to monitor data quality and potential contamination.

### 2.5 Library preparation and shotgun metagenomic sequencing

The DNA libraries were prepared using the Illumina DNA Prep (M) Tagmentation Kit for 96 samples (Illumina, Cat. No. 20049006, San Diego, CA, United States) and IDT for Illumina DNA/RNA Unique Dual Indexes (Illumina, Cat. No. 20026121, San Diego, CA, United States), following the manufacturer’s protocol. Approximately 100 ng of total microbial DNA per-sample was quantified using a Qubit fluorometer (Thermo Fisher Scientific, Waltham, MA, United States of America). Library preparation involved bead-linked transposome-mediated tagmentation, which simultaneously fragmented the DNA and added adapter sequences. Post-tagmentation, limited-cycle PCR amplification (10 cycles) was performed to incorporate index adapters. Libraries were dual-sided size-selected using AMPure XP bead cleanup (Beckman Coulter, Brea, CA, United States) to enrich fragments around ∼450 bp. Fragment size distribution was assessed using the Agilent TapeStation System (Agilent Technologies, Santa Clara, CA, United States). Libraries were normalized to 4 nM, pooled equimolarly, and diluted to a final loading concentration of 750 pM (∼0.75 ng/μL). Sequencing was performed on the Illumina NextSeq 2000 platform using a 2 × 300 bp paired-end run configuration with the P2 XLEAP-SBS™ 600-cycle reagent kit at KAIMRC. A 1% PhiX Control (Illumina, Cat. No. 15017872) was spiked in to monitor sequencing performance. Demultiplexing and initial quality filtering of paired-end reads were performed prior to downstream bioinformatics analysis. Libraries were prepared and sequenced in two runs with samples randomized by age group. Each run included positive and negative controls processed alongside the study samples.

### 2.6 Bioinformatics and statistical analyses

For fecal microbiome analyses, raw FASTQ files were trimmed and filtered using fastp (v1.0.1) with adapter detection and sliding-window quality trimming (Chen et al., 2018). Human host-genomes were removed by alignment to the reference genome (GRCh38) using Bowtie2 (v2.5.4) (Langmead and Salzberg, 2012; Schneider et al., 2017). After quality control, the mean per-sample sequencing depth was 15×, and base-quality metrics indicated more than 98.8% ± 0.6%.

Taxonomic profiles were generated with MetaPhlAn (v4.2.2) using the CHOCOPhlAn 4 marker database (build mpa_v[Jan25] _CHOCOPhlAnSGB, released [202,503]), which uses clade-specific marker genes to estimate relative abundances at the phylum and species levels. For inferential analyses, a single cohort-level feature set was defined *a priori*: taxa detected in ≥10% of samples with a mean relative abundance of ≥0.1% across the cohort. This set was used for all between-group comparisons to avoid group-specific selection bias. Taxa with ≥1% relative abundance in at least one sample were used for descriptive summaries. Core taxa within each age group were defined as present in ≥50% of samples with a mean relative abundance ≥0.1%; the overall SAMS core was the union across groups (G1–G5). All bioinformatics analyses were conducted on a Linux-based high-performance computing environment at KAIMRC. All scripts are available on GitHub: (https://github.com/KAIMRC-DAIB/biomage.git).

All statistical analyses were performed in R (v4.5.1). Baseline metadata were summarized to describe the study population. Continuous variables were presented as mean ± standard deviation (SD), with corresponding 95% confidence intervals (CIs) where applicable, and were compared between groups using the Kruskal–Wallis test. Categorical variables were presented as counts and percentages (n, %) with 95% CIs for proportions, and were compared using chi-square or Fisher’s exact tests, as appropriate. All multivariable analyses incorporated biologically relevant covariates, including sex, body mass index (BMI), job status, family size, socialization status, smoking, sleep difficulty, and supplement use, based on previous literature (Qian et al., 2020; Inoue et al., 2024; Batalha et al., 2025). Relative abundance data expressed as percentages (%), representing compositional proportions, retaining zero values without imputation or pseudocount addition, and no log or centered log-ratio (CLR) transformation was applied. The α-diversity indices (observed richness, Shannon, Simpson, and Pielou’s evenness) were computed from the MetaPhlAn relative abundance profiles using the vegan package (v2.7–1). Multivariable linear regression models were used to investigate associations between age groups and alpha diversity indices (Shannon, Simpson, observed richness, and Pielou’s evenness), adjusted for the covariates described above. Pairwise comparisons between groups were estimated using marginal means implemented in the emmeans package. The β-diversity was assessed using the Bray–Curtis dissimilarities on species-level relative abundances (vegdist, vegan, v2.7–1) and visualized by principal coordinates analysis (PCoA). Differences in community composition were tested using permutational multivariate analysis of variance (PERMANOVA; adonis2, 999 permutations) with adjustment for the predefined covariates. Effect sizes were summarized using *R* ^2^ values. Pairwise PERMANOVA comparisons between groups were performed, with p-values adjusted for multiple testing using Benjamini–Hochberg false discovery rate (BH-FDR). Homogeneity of group dispersion was assessed using PERMDISP to confirm the appropriateness of PERMANOVA. Statistical significance was determined using BH-FDR adjusted *q*-values. Figures annotate BH-FDR-adjusted q-values and corresponding significance asterisks. For positive control, concordance between observed and manufacturer-expected profiles (Zymo Research, Cat. No. D6300, Irvine, CA, United States of America) was quantified using Bray–Curtis dissimilarity and Pearson correlation on relative abundances. For taxonomic comparisons, relative abundances were analyzed across age groups using confounder-adjusted linear regression models at the phylum and species levels. To limit multiplicity, analyses were restricted to phyla with cohort-wide mean ≥ 1% and the top 30 species by mean relative abundance ≥1%. Effect sizes were summarized using regression slopes and fold changes between the oldest and youngest age groups, and statistical significance was determined using BH-FDR-adjusted q-values (q < 0.05). Bootstrap resampling (1,000 iterations) was used to derive 95% confidence intervals for Spearman correlation coefficients. Partial Spearman correlations adjusted for all selected covariates were additionally computed, with p-values corrected using BH-FDR. Age–taxon associations shown in figures were required to meet a two-sided FDR-adjusted q < 0.05. Figure selection for age-taxon plots used two-sided FDR-adjusted *q* ≤ 0.05, and species panels were limited to the 30 taxa with the highest mean relative abundance.

## 3 Results

### 3.1 Saudi aging and microbiome study (SAMS): cohort characteristics

This study analyzed fecal metagenomes from 145 healthy Saudi adults aged 19–69 years. As detailed in Table 1, participants were categorized into five age groups: G1 (19–29 years, n = 33; 22.7%), G2 (30–39 years, n = 30; 20.7%), G3 (40–49 years, n = 27; 18.6%), G4 (50–59 years, n = 31; 21.4%), and G5 (60–69 years, n = 24; 16.6%). The mean age was 42.7 ± 14 years (95% CI: 40.4–45). Sex distribution did not differ significantly among age groups (Fisher’s exact*, p* = 0.948), as (75/145; 51.7%; 95% CI: 43.6–59.9) were males and (70/145; 48.3%; 95% CI: 40.1–56.4) were females.

**TABLE 1:** Demographic and lifestyle characteristics of the Saudi Aging and Microbiome Study (SAMS) pilot cohort.

Parameter	Total n = 145 (100%)	Group 1 19–29 n = 33 (22.7%)	Group 2 30–39 n = 30 (20.7%)	Group 3 40–49 n = 27 (18.6%)	Group 4 50–59 n = 31 (21.4%)	Group 5 60–69 n = 24 (16.6%)	P value
A. Demographics							
Age, years-mean ±SD	42.7 ± 14	24.5 ± 2.8	34.2 ± 3	43.5 ± 3	54.2 ± 3.6	62.7 ± 2.5	<0.001
BMI, kg/m2-mean ±SD	26.3 ± 4.2	25.2 ± 4.6	26.7 ± 5.2	26.6 ± 4.1	26.2 ± 3.3	27.1 ± 3.4	0.137
Sex, n (%)							
Female	70 (48.3%)	17 (51.5%)	14 (46.7%)	14 (51.9%)	15 (48.4%)	10 (41.7%)	0.948
Male	75 (51.7%)	16 (48.5%)	16 (53.3%)	13 (48.1%)	16 (51.6%)	14 (58.3%)	
Family size, n (%)							
Small	36 (24.8%)	5 (15.2%)	16 (53.3%)	9 (33.3%)	1 (3.2%)	5 (20.8%)	<0.001
Medium	46 (31.7%)	9 (27.3%)	5 (16.7%)	7 (25.9%)	19 (61.3%)	6 (25%)	
Large	63 (43.4%)	19 (57.6%)	9 (30%)	11 (40.7%)	11 (35.5%)	13 (54.2%)	
B. Socioeconomic status							
Job status, n (%)							
Employee	126 (86.9%)	33 (100%)	30 (100%)	27 (100%)	31 (100%)	5 (20.8%)	<0.001
Retirement	19 (13.1%)	0 (0%)	0 (0%)	0 (0%)	0 (0%)	19 (79.2%)	
C. Lifestyle and physical activity							
Sleep difficulty, n (%)	36 (24.8%)	5 (15.2%)	4 (13.3%)	7 (25.9%)	15 (48.4%)	5 (20.8%)	0.0102
Social activity, n (%)							
Low	26 (17.9%)	10 (30.3%)	8 (26.7%)	4 (14.8%)	4 (12.9%)	0 (0%)	0.00373
Medium	64 (44.1%)	14 (42.4%)	12 (40%)	17 (63%)	14 (45.2%)	7 (29.2%)	
High	55 (37.9%)	9 (27.3%)	10 (33.3%)	6 (22.2%)	13 (41.9%)	17 (70.8%)	
Exercise, n (%)	95 (65.5%)	23 (69.7%)	18 (60%)	16 (59.3%)	17 (54.8%)	21 (87.5%)	0.0944
Outside	89 (61.4%)	23 (69.7%)	17 (56.7%)	14 (51.9%)	15 (48.4%)	20 (83.3%)	
Inside	6 (4.1%)	0 (0%)	1 (3.3%)	2 (7.4%)	2 (6.5%)	1 (4.2%)	
Smokers, n (%)	17 (11.7%)	0 (0%)	7 (23.3%)	5 (18.5%)	4 (12.9%)	1 (4.2%)	0.0128
D. Supplement use							
Vitamins, n (%)	62 (42.8%)	10 (30.3%)	14 (46.7%)	7 (25.9%)	13 (41.9%)	18 (75%)	0.0036
Omega-3, n (%)	22 (15.2%)	4 (12.1%)	2 (6.7%)	1 (3.7%)	4 (12.9%)	11 (45.8%)	<0.001
Zinc, n (%)	10 (6.9%)	3 (9.1%)	6 (20%)	0 (0%)	1 (3.2%)	0 (0%)	0.0137
Prebiotic, n (%)	1 (0.7%)	1 (3%)	0 (0%)	0 (0%)	0 (0%)	0 (0%)	1
Probiotic, n (%)	4 (2.8%)	1 (3%)	1 (3.3%)	0 (0%)	2 (6.5%)	0 (0%)	0.776

Abbreviations: BMI, body mass index. Continuous variables are presented as mean ± SD, and categorical variables as n (%). Between-group comparisons were performed using Kruskal–Wallis tests for continuous variables and two-sided Fisher’s exact tests for categorical variables. Corresponding 95% confidence intervals are provided in Supplementary Table S1. Analyses were conducted in R (v4.5.1).

The cohort health status was consistent with the established health criteria across adult groups including older participants as defined by the World Health Organization and the National Institute on Aging and described in the Methods Section (NIA, 2022; WHO, 2022). The mean body mass index (BMI) of the study participants was 26.3 ± 4.2 kg/m^2^ (95% CI: 25.6–27), with no significant differences across age groups (*p* = 0.948). Most participants reported regular physical activity (95/145; 65.5%; 95% CI: 57.8–73.3), and no significant differences in pet ownership were observed across age groups (*p* > 0.05). The use of prebiotics and/or probiotics was uncommon (1/145; 0.7%; 95% CI: 0–2, and 4/145; 2.8%; 95% CI: 0.1–5.4, respectively) and did not differ significantly across the age groups (*p* > 0.05). Most participants were employed, married, non-smokers, lived in medium-to large-sized households, and reported moderate to high levels of social activity. Detailed distributions of all demographics, lifestyle, and clinical variables, including 95% confidence intervals are provided in Supplementary Table S1.

### 3.2 Alpha and beta diversity among different age groups in healthy adults

The microbial taxonomic composition was profiled from 145 fecal samples sequenced by shotgun metagenomics using the Illumina NextSeq 2000 platform. Each sequencing run included two quality control (QC) libraries. After QC and host-read removal, sequencing yielded a total of 870.9 GB data and 1,534,300,000 total reads, with a means of 5.92 ± 6.67 GB and 10.4 ± 12.8 million reads per library. The observed composition of the mock communities matched the manufacturer’s expected profile (Bray–Curtis 0.145 and 0.119; Pearson r = 0.871 and 0.883 across runs), and negative controls were below the detection threshold (Supplementary Figure S1). The microbial taxonomic profiles were generated with MetaPhlAn-4 and were used to analyze the α- and β-diversity, to characterize the fecal microbial composition at the phylum and species levels among age groups, and to identify the age-associated microbial patterns, including the core fecal microbiome.

**FIGURE 2:**
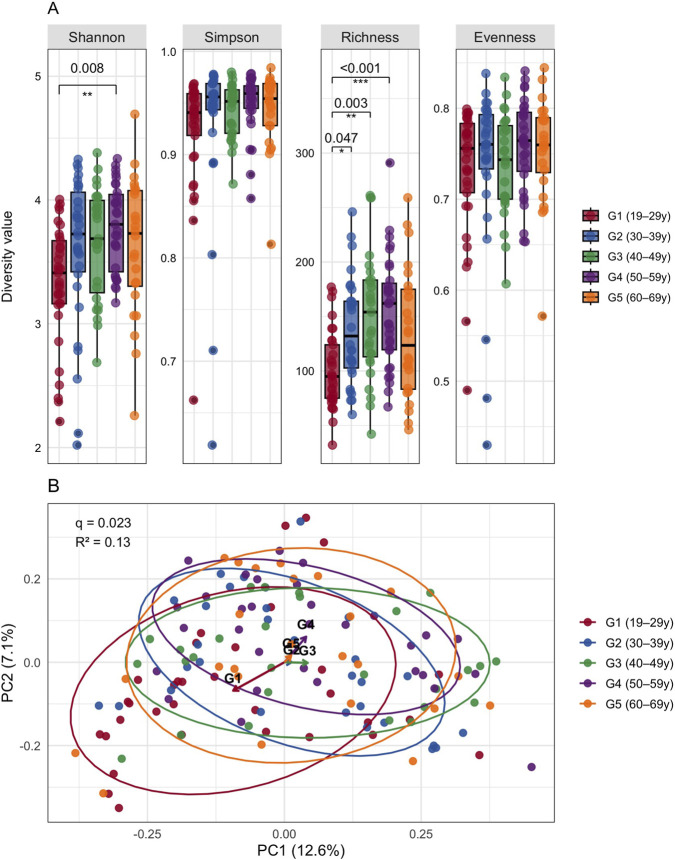
Microbial alpha and beta diversity among age groups in healthy adults. **(A)** Boxplots display the distribution of α-diversity metrics (Shannon, Simpson, observed richness, Pielou’s evenness) calculated at the species level across five age groups (G1-G5; 19–69 years). Differences across age groups were evaluated using multivariable linear regression models adjusted for predefined covariates. Pairwise comparisons were estimated using marginal means, and statistical significance was determined using Benjamini–Hochberg false discovery rate (BH–FDR)–adjusted q-values. **(B)** Principal coordinates analysis (PCoA) of Bray–Curtis dissimilarities calculated at the species level illustrates between-group β-diversity. Differences in overall community composition were tested using permutational multivariate analysis of variance (PERMANOVA; adonis2, 999 permutations); the panel reports the BH–FDR–adjusted q-value and effect size (*R* ^*2*^ *).* Significance is indicated by asterisks.

After adjustment for relevant confounders, α-diversity differed across age groups in a metric-specific manner (Figure 2A). Observed richness, which reflects the number of detected taxa, was significantly higher in G2, G3, and G4 compared with G1(BH-FDR–adjusted *q* = 0.047, 0.003, 0.001), respectively. Shannon diversity, which accounts for both taxon richness and evenness, was significantly higher in G4 compared with G1 (BH-FDR–adjusted *q* = 0.008). In contrast, Simpson diversity, which emphasizes dominance by abundant taxa, and Pielou’s evenness, which quantifies how evenly taxa are distributed within a community, did not differ significantly among age groups after multiple-testing correction (all q > 0.05). Overall, the α-diversity of the fecal microbiome increased from young to middle adulthood, then remained relatively stable in the older age groups.

Principal coordinates analysis (PCoA) based on Bray–Curtis dissimilarities was used to assess between-sample variation in the fecal microbiome composition (Figure 2B). Global permutational multivariate analysis of variance (PERMANOVA) suggested a significant overall effect of age group on community structure (*R* ^*2*^ = 0.13, BH–FDR–adjusted *q* = 0.023). However, pairwise PERMANOVA comparisons between age groups did not differ significantly after BH-FDR correction (all q > 0.05; Supplementary Table S2), although minor, non–FDR-significant differences were observed for G1 versus G2 (p = 0.008, *q* = 0.08; *R* ^2^ = 0.228) and G1 versus G3 (p = 0.019, *q* = 0.095; *R* ^2^ = 0.236). These results suggest that age-related shifts in overall community structure are detectable at the global level but are not resolved in specific pairwise contrasts after multiple-testing correction.

### 3.3 Fecal microbial composition at the phylum and species levels across different age groups in healthy adults

**FIGURE 3:**
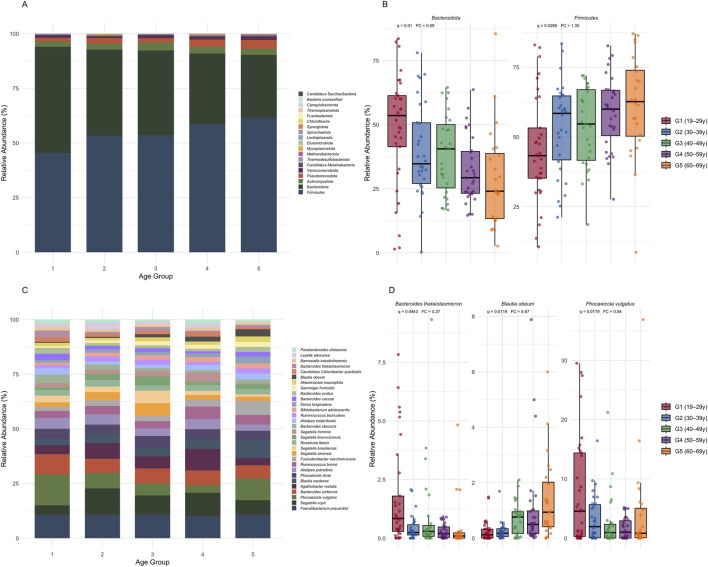
Fecal microbiome composition across age groups in healthy adults. **(A)** Stacked bar plots showing the mean relative abundances of all detected phyla among the five healthy age groups. **(B)** Box plots depicting dominant phyla exhibiting significant age-associated differences based on multivariable linear regression analyses. **(C)** Stacked bar plots showing the 30 most abundant species across age groups. **(D)** Box plots depicting species exhibiting significant age-associated differences based on multivariable linear regression analyses. Statistical significance was determined using Benjamini–Hochberg false discovery rate (BH–FDR)–adjusted q-values (q < 0.05).

The fecal microbiome was profiled across the five age groups (G1-G5) at the phylum and species levels (Figure 3). Age-related changes in taxon relative abundance were assessed using multivariable linear regression models adjusted for sex, BMI, job status, family size, social activity, smoking, sleep difficulty, vitamin use, omega-3 use, and zinc supplementation.

The phylum-level analyses were restricted to taxa with a cohort-wide mean relative abundance ≥1% (Figure 3A). In total, 18 phyla met this criterion, with observed shifts in the relative abundance across the age groups (G1-G5). The relative abundance of the dominant phyla that changed significantly with age were *Firmicutes* and *Bacteroidota* (formerly *Bacteroidetes*) (Figure 3B). Specifically, the relative abundance of *Firmicutes* increased significantly with age from G1 to G5 (FC = 1.35; *q* = 0.0265), indicating a 35% higher adjusted relative abundance in older adults, whereas the relative abundance of *Bacteroidota* decreased significantly with age, from G1 to G5 (FC = 0.59; *q* = 0.01), corresponding to a 41% lower adjusted relative abundance in older adults. Together, these opposing linear trends suggest a pronounced age-related restructuring of the dominant taxonomic composition of the fecal microbiome.

At the species level, analyses were restricted to the 30 most abundant species determined by a cohort-wide mean relative abundance (≥1%) across the age groups (Figure 3C). Among these, three species differed significantly with age after BH-FDR correction: *Bacteroides thetaiotaomicron, Phocaeicola vulgatus*, and *Blautia obeum* (Figure 3D). *B. thetaiotaomicron* was the most abundant species in the youngest age group (G1) and declined significantly in the older age groups (G5) (FC = 0.27; *q* < 0.0443), corresponding to a 73% lower adjusted relative abundance in older adults. Similarly, *P. vulgatus* showed a significant decrease with age (FC = 0.54; *q* < 0.0119), corresponding to a 46% lower adjusted relative abundance in G5 relative to G1. However, *B*. *obeum* exhibited a significant age-related increase, with a 5.67-fold higher adjusted relative abundance in G5 compared to G1 (FC = 5.67; *q* < 0.0119). These findings suggest restructuring of the fecal microbial community with chronological age.

### 3.4 Identification of the core fecal microbiome among different age groups in healthy adults

**FIGURE 4:**
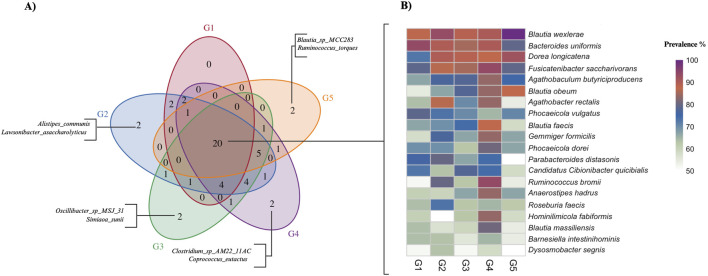
The core gut microbiome among age groups in the SAMS healthy adult cohort (G1-G5). **(A)** Venn diagram of core species shared among five age groups (G1-G5). Core taxa are defined as species with relative abundance ≥0.1% in ≥50% of samples within a given group. Species unique to each age group are labeled outside the corresponding circle. **(B)** Heatmap of core species shared among age groups. Cell color encodes prevalence (%) within each group, calculated as the fraction of samples in which a species reaches ≥0.1% relative abundance. The right-hand color bar indicates the prevalence scale.

The core fecal microbiome was determined within each age group as species detected in ≥50% of the samples with the mean relative abundance >0.1%, following established thresholds described previously (Neu et al., 2021; Mancabelli et al., 2024). To characterize the representative microbial species among the age groups, the intersections of group-specific core and non-core “accessory” sets were identified (Figure 4). Figure 4A illustrates the number of shared core species among the age groups and those unique core species for each age group. G1 (19–29 y) did not display exclusive core species, whereas G2 to G5 each had two group-specific taxa. For instance, G2 (30–39 y) had *Alistipes communis* and *Lawsonibacter asaccharolyticus*, G3 (40–49 y) had *Oscillibacter* sp. *MSJ_31* and *Simiaoa sunii*, G4 (50–59 y) had *Clostridium* sp. *AM22_11AC* and *Coprococcus eutactus*, and G5 (60–69 y) had *Blautia* sp. *MCC283* and *Ruminococcus torques*. These microbial patterns suggest an increasing age-related differentiation of the fecal microbiome, consistent with the beta-diversity trend that showed more distinct community structures in the older adults. Figure 4B shows the 20 species that were core among all age groups. Four of these species, consisting of *Blautia wexlerae*, *Bacteroides uniformis*, *Dorea longicatena*, and *Fusicatenibacter saccharivorans*, exhibited ≥70% prevalence in each age group.

### 3.5 Age-associated trends in the fecal microbiome composition in healthy adults

**FIGURE 5:**
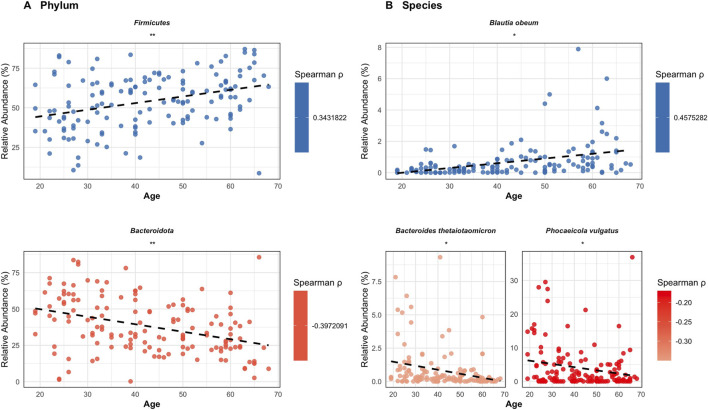
Correlations between fecal microbial taxa and chronological aging among healthy adult individuals. **(A)** Phylum-level and **(B)** species-level results. Points represent taxa showing significant partial Spearman correlations with chronological age after adjustment for predefined confounders and computed, and p-values were corrected using the Benjamini–Hochberg false discovery rate (BH–FDR). Colors indicate direction (blue, positive; red, negative) and point saturation scales with the magnitude of the correlation (|ρ|), as shown by the color bar. Dashed lines depict ordinary least-squares (OLS) fits for visualization only. Significance is indicated by asterisks.

Correlations between age and the microbial relative abundance across healthy adults were assessed using Spearman’s rank correlation and confounder-adjusted partial Spearman correlation analyses with p-values corrected using BH-FDR (Figure 5; Supplementary Table S5). Two bacterial phyla and three species showed significant correlations with chronological age after multiple-testing correction.

At the phylum level (Figure 5A), *Firmicutes* showed a significant positive correlation with chronological age (Spearman’s ρ = 0.343, 95% CI 0.18 to 0.49, q = 0.006), indicating an increase in relative abundance with aging in healthy adults. In contrast, *Bacteroidota* (formerly *Bacteroidetes*) showed a significant negative correlation with chronological age (Spearman’s ρ = −0.397, 95% CI -0.546 to −0.238, *q* = 0.002), reflecting a progressive decline in relative abundance across the healthy lifespan.

At the species level (Figure 5B), *B. obeum* demonstrated a significant positive correlation with aging (Spearman’s ρ = 0.457, 95% CI 0.311 to 0.579, *q* = 0.02), indicating an increase in relative abundance in healthy older individuals. Conversely, *B. thetaiotaomicron* showed a significant negative correlation with aging (Spearman’s ρ = −0.349, 95% CI -0.498 to −0.189, *q* = 0.02), consistent with reduced abundance in older age groups. *P. vulgatus* exhibited a negative trend with aging (Spearman’s ρ = −0.17, 95% CI -0.329 to 0.011, *q* = 0.02), although the confidence interval slightly overlapped zero, suggesting borderline significance.

## 4 Discussion

To the best of our knowledge, this study provides the first characterization of age-associated changes in the fecal microbiome across healthy adults in the Gulf region. In this Saudi Aging and Microbiome Study (SAMS), fecal samples from 145 healthy adults living in Riyadh and aged 19 to 69 were profiled to identify the potential microbial taxa associated with chronological age in healthy adult populations. While several studies from the Middle East have described gut microbiome patterns in children or adult populations with specific diseases, no prior investigations have specifically examined the age-associated gut microbiome variation in a healthy adult cohort in this region (Plummer et al., 2020; Alzahrani, 2021). Further, previous studies have described age-related shifts in fecal microbiome composition, often without a clear definition of healthy aging in older adult populations (Li et al., 2021; Wilmanski et al., 2021). In the SAMS cohort, we defined health status for adults and healthy aging for older adults aged 60 years and above according to the WHO and NIA criteria (NIA, 2022; WHO, 2022), requiring preserved cognitive ability, functional independence, and active social engagement, as detailed in the Methods Section. The presented healthy cohort profile assists in focusing on age-related taxa that are potentially associated with healthy aging.

Shotgun metagenomics profiling revealed a higher alpha diversity in the older adults than in the younger, along with age-associated variation in β-diversity as assessed by Bray–Curtis dissimilarities. Although the global PERMANOVA test suggested an overall age effect, pairwise comparisons between individual age groups did not remain significant after FDR correction. Similar diversity findings were reported in aging studies, including very young to healthy older adult populations (Odamaki et al., 2016; Mancabelli et al., 2024). Despite the fact that the present cohort does not include individuals of extreme old age, similar directional patterns were reported in studies of nonagenarians and centenarians, in which increased microbial diversity and age-related shifts in overall community composition were observed (Xu et al., 2022). Nevertheless, a previous study indicated contrasting trends (Ravikrishnan et al., 2024; Litichevskiy et al., 2025), which suggest that the diversity direction and association with health outcomes might vary across cohorts, microbial features, and host contexts.

Across the healthy age groups, the fecal microbial community was predominantly composed of *Firmicutes* and *Bacteroidota*, consistent with the typical phylum-level structure of the healthy adult gut microbiome (Maynard and Weinkove, 2018). Aging in the context of frailty or disease has often been associated with increased *Bacteroidota* and *Proteobacteria*, and reduced *Firmicutes*, a pattern described as gut dysbiosis (Maynard and Weinkove, 2018). However, with increasing age among healthy adults, we observed an increase in *Firmicutes* accompanied by a progressive decline in *Bacteroidota,* resulting in an increased *Firmicutes*/*Bacteroidota* (F/B) ratio. This pattern is consistent with previous population-based studies that examined the fecal microbiome of healthy adults, older adults, and centenarians (Odamaki et al., 2016; Kim et al., 2019; Ai et al., 2025). A higher relative abundance of *Firmicutes* is commonly associated with increased production of short-chain fatty acids (SCFAs), particularly butyrate and propionate, which promote gut barrier integrity, regulate immune function, and support host metabolic health (Vaiserman et al., 2020). Together, these findings suggest that the microbial diversity and composition in healthy Saudi adults follow trajectories broadly consistent with those observed in other populations. The current results also suggest that the fecal microbiome and healthy aging may be conserved across different geographic and cultural settings.

Several microbial species identified in our analyses have well-established functional roles relevant to host health across the adult lifespan, particularly through their involvement in SCFAs metabolism. Species within the genera *Blautia*, *Bacteroides*, and *Phocaeicola* contribute to carbohydrate fermentation pathways that generate key SCFAs such as acetate, propionate, and butyrate, metabolites known to support gut barrier integrity, regulate immune responses, and influence metabolic biomarkers linked to healthy aging, as reported in previous studies (Louis and Flint, 2017; Liu et al., 2021).

At the species level, increasing chronological age among healthy adults in the SAMS pilot cohort was positively correlated with *B. obeum*. *B. obeum* (formerly *Ruminococcus obeum*) is involved in SCFAs production, particularly acetate and butyrate, which play key roles in maintaining gut barrier integrity and regulating immune homeostasis (Ragonnaud and Biragyn, 2021). The positive correlation and increased abundance of *B*. *obeum* observed in our cohort align with recent findings that have linked reduced *B*. *obeum* levels to neurological disorders and cognitive decline, suggesting that this species may contribute to neurocognitive health across adult populations (Rout et al., 2025).

In contrast, this pilot study identified two species within the phylum *Bacteroidota*, *B. thetaiotaomicron* and *P. vulgatus* (formerly *Bacteroides vulgatus*), that were negatively correlated with chronological age among healthy adults. *B. thetaiotaomicron* is a core species of fecal microbiome and has been associated with reduced frailty and preserved physiological function in some populations (Forster et al., 2019; Mancabelli et al., 2024). However, despite these reported benefits, its relative abundance decreased with age in our healthy adult cohort, suggesting that even in the absence of chronic diseases, aging is accompanied by a remodeling of key microbial functions. *B*. *thetaiotaomicron* is a major polysaccharide-degrading bacterium in the human gut, characterized by a diverse repertoire of carbohydrate-active enzymes that enable extensive fermentation of complex carbohydrates into bioactive metabolites such as SCFAs, thereby influencing host energy metabolism, immune regulation, and gut-host crosstalk (Ye et al., 2021). Moreover, *B. thetaiotaomicron* has been linked to reduced frailty and diminished inflammaging (Le Cosquer et al., 2024). Its negative correlation with aging in our dataset suggests potential context-dependent effects that might be influenced by diet, geography, and strain composition variation.

We also observed a significant age-associated decline in the relative abundance of *P*. *vulgatus*. This species exhibited a negative correlation with chronological age, suggesting that it becomes less dominant in the gut microbiome as individuals age. Consistent with this finding, a previous study examined different age ranges reported a lower abundance of *P*. *vulgatus* in healthy older adults and centenarians compared with younger adults (Park et al., 2015). However, a more recent study reported a higher abundance of *P*. *vulgatus* in physically active older adults, suggesting a potential context-dependent role (Fiorilli et al., 2025). *P*. *vulgatus* has received limited attention in literature which necessitates further research to clarify the functional implications of this bacterium decline and its potential relevance to host physiology in later life. These species-level patterns presented here were derived from continuous-age analyses using both Spearman and confounder-adjusted partial Spearman correlation models, suggesting that the observed trends reflect gradual microbial changes across adulthood rather than effects driven solely by categorical age-group comparisons.

The present pilot study identified a healthy, age-related core microbiome, defined as species present in ≥50% of samples at ≥0.1% mean relative abundance among all adult age groups, resulted in 20 species (Supplementary Figure S2; Supplementary Tables S3, S4). In comparison with the previously reported 56-species of the global core fecal microbiome derived from pooled metagenomic sequencing data across 37 country-level cohorts (Mancabelli et al., 2024), 12 (60%) of SAMS species overlapped. However, eight (40%) of SAMS species (*Barnesiella intestinihominis*, *Blautia faecis*, Candidatus *Cibionibacter quicibialis*, *Dysosmobacter segnis*, *Fusicatenibacter saccharivorans*, *Hominilimicola fabiformis*, *Roseburia faecis*, and *Ruminococcus bromii*) appeared to be unique to the Saudi cohort, suggesting a substantial overlap and distinct regional fecal microbiome signature. From a biological perspective, *B. intestinihominis* has been proposed as an immunomodulatory probiotic in younger Chinese populations (Yan et al., 2022), Candidatus*. C. quicibialis* may contribute to vitamin B12 (cobalamin) synthesis (Grønbæk et al., 2025), and *D. segnis* has been linked to favorable lipid metabolism (Petitfils et al., 2025). Several core members, *B*. *faecis*, *H. fabiformis*, *R*. *faecis*, and *R*. *bromii*, are established producers or promoters of SCFAs, particularly butyrate, via carbohydrate fermentation and cross-feeding. This SCFAs-enriched, immunomodulatory profile may help preserve gut epithelial barrier integrity, regulate inflammation, and maintain metabolic homeostasis (Warman et al., 2022; Choi et al., 2023; Van den Abbeele et al., 2023; Song et al., 2024). Additionally, *F*. *saccharivorans* may reduce intestinal inflammation by stimulating anti-inflammatory mediator production (Mai et al., 2022). Collectively, the persistence of these taxa across adults suggests that Saudi adults harbor a core microbiome profile that may contribute to resilience against age-related inflammatory and metabolic decline and may be amenable to dietary, prebiotic, or targeted probiotic strategies.

This pilot study identified the first comprehensive analysis of the fecal microbiome in healthy adults in Saudi Arabia. It expands the geographic scope of research on age-related changes in the gut microbiome in healthy populations. Shotgun metagenomics technology implemented here provided higher species-level taxonomic resolution than 16S rRNA profiling, and the age-grouped design enabled assessment of diversity, definition of a core microbiome, and detection of chronological age-related compositional shifts across healthy adults of different ages. As a pilot phase, SAMS was designed to focus on healthy individuals in order to establish a rigorous, region-specific microbiome reference under strict health criteria, thereby creating a foundational framework for subsequent multi-omics and disease-focused investigations. Future phases of SAMS will expand recruitment to include age-matched individuals with chronic age-associated disease, enabling direct comparisons with the healthy reference cohort and enhancing the translational relevance of this work.

A limitation of this study is the absence of direct functional pathway reconstruction (e.g., HUMAnN-based metabolic profiling). Future phases of the SAMS cohort will incorporate comprehensive metagenomic functional analyses at the metabolic pathway level for the microbial taxa reported in this study to determine their metabolic capacity more accurately. Nevertheless, this pilot phase provided an important opportunity to evaluate the feasibility and applicability of the taxonomic analytical framework that will underpin functional investigations in subsequent studies. The current methodology consistently identified age-associated taxonomic patterns that are concordant with prior reports in healthy populations, supporting the robustness of the current analytical workflow and the biological possibility of the future functional analyses.

The current age range (19–69 years) captures age-related microbiome variation within healthy young and older adults rather than the full spectrum of late-life healthy aging or longevity. Hence, future studies building on this pilot cohort will expand recruitment to include more advanced age groups (Figure 1). Although technical variability was minimized through standardized DNA extraction and library preparation, and by including mock communities and negative controls in each sequencing run; minor residual batch effects cannot be fully excluded. The cohort was geographically localized and relatively homogeneous in socioeconomic background, which may limit the generalizability of the findings to more diverse populations.

## 5 Conclusion

This pilot study provides a shotgun-metagenomic baseline data of the healthy adult fecal microbiome in Saudi Arabia and defines its age-related variation. We observed increasing diversity and compositional shifts with chronological age that aligned with global patterns and identified the adult core microbiome. This study reported that multiple taxa showed significant correlation with chronological age among healthy adults. These findings collectively revealed a predominantly conserved pattern of age-associated microbiome, characterized by Saudi-specific features that may enhance resilience in later life.

## Data Availability

The sequencing data supporting this study are available through SRA under study accession number PRJNA1356643.
