# Supplementary material for: First metagenomic analysis of age-associated changes in the gut microbiome among healthy Saudi adults: SAMS pilot study
Source: Front Aging. 2026 Mar 11;7:1733638. doi: 10.3389/fragi.2026.1733638 (PMC13013359; doi:10.3389/fragi.2026.1733638)
Supplement: Supplementary file 1 [file Supplementaryfile1.docx]

Supplementary Material

# Supplementary Figures and Tables

## Supplementary Figures


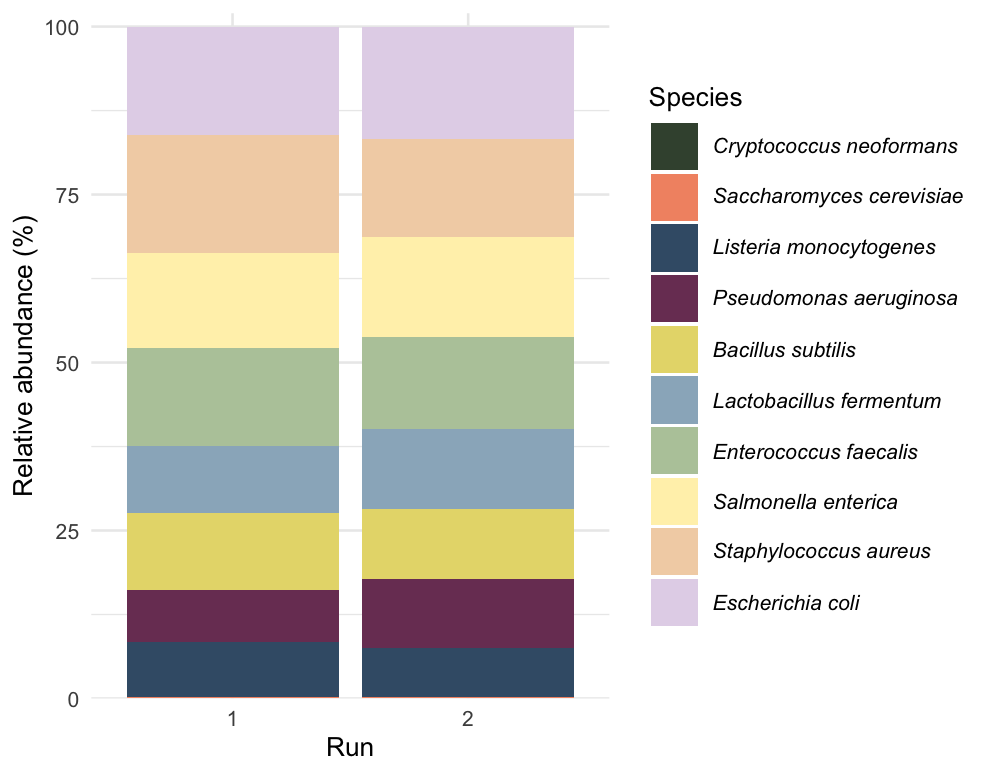


**Supplementary Figure 1.** **Validation of the positive controls across sequencing runs.** The relative abundance profiles of the ZymoBIOMICS™ microbial community standard are presented from two different sequencing runs. The y-axis defines relative abundance, whereas the x-axis corresponds to the run number. Positive controls were processed alongside the study samples in each run. The observed composition of the community aligned with the ZymoBIOMICS™ reference profile from the manufacturer, ensuring reproducibility and data quality across runs.

Table S1: Extended participant characteristics with 95% confidence intervals stratified by age group.

| **Parameter** | **Total** n=145 (100%) | **Group 1 19–29** n=33 (22.7%) | **Group 2 30–39** n=30 (20.7%) | **Group 3 40–49** n=27 (18.6%) | **Group 4 50–59** n=31 (21.4%) | **Group 5 60–69** n=24 (16.6%) | **P-value** |  |
| --- | --- | --- | --- | --- | --- | --- | --- | --- |
| **Age**, years-mean ±SD, (95% CI) | 42.7 ± 14 (40.4-45) | 24.5 ± 2.8 (23.5-25.5) | 34.2 ± 3 (33.1-35.3) | 43.5 ± 3 (42.4-44.6) | 54.2 ± 3.6 (52.9-55.5) | 62.7 ± 2.5 (61.7-63.7) | <0.001 |  |
| **BMI**, kg/m2-mean ±SD, (95% CI) | 26.3 ± 4.2 (25.6-27) | 25.2 ± 4.6 (23.6-26.8) | 26.7 ± 5.2 (24.8-28.6) | 26.6 ± 4.1 (25.1-28.1) | 26.2 ± 3.3 (25-27.4) | 27.1 ± 3.4 (25.7-28.5) | 0.137 |  |
| **Sex,** n (%), **(95% CI)** |  |  |  |  |  |  |  |  |
| Female | 70 (48.3%), (40.1-56.4) | 17 (51.5%), (34.5-68.6) | 14 (46.7%), (28.8-64.5) | 14 (51.9%), (33-70.7) | 15 (48.4%), (30.8-66) | 10 (41.7%), (21.9-61.4) | 0.948 |  |
| Male | 75 (51.7%), (43.6-59.9) | 16 (48.5%), (31.4-65.5) | 16 (53.3%), (35.5-71.2) | 13 (48.1%), (29.3-67) | 16 (51.6%), (34-69.2) | 14 (58.3%), (38.6-78.1) |  |  |
| Job status, n (%) |  |  |  |  |  |  |  |  |
| Employee | 126 (86.9%), (81.4-92.4) | 33 (100%), (100-100) | 30 (100%), (100-100) | 27 (100%), (100-100) | 31 (100%), (100-100) | 5 (20.8%), (4.6-37.1) | <0.001 |  |
| Retirement | 19 (13.1%), (7.6-18.6) | 0 (0%), (NA) | 0 (0%), (NA) | 0 (0%), (NA) | 0 (0%), (NA) | 19 (79.2%), (62.9-95.4) |  |  |
| **Family size,** n (%), (95% CI) |  |  |  |  |  |  |  |  |
| Small | 36 (24.8%), (17.8-31.9) | 5 (15.2%), (2.9-27.4) | 16 (53.3%), (35.5-71.2) | 9 (33.3%), (15.6-51.1) | 1 (3.2%), (0-9.4) | 5 (20.8%), (4.6-37.1) | <0.001 |  |
| Medium | 46 (31.7%), (24.1-39.3) | 9 (27.3%), (12.1-42.5) | 5 (16.7%), (3.3-30) | 7 (25.9%), (9.4-42.5) | 19 (61.3%), (44.1-78.4) | 6 (25%), (7.7-42.3) |  |  |
| Large | 63 (43.4%), (35.4-51.5) | 19 (57.6%), (40.7-74.4) | 9 (30%), (13.6-46.4) | 11 (40.7%), (22.2-59.3) | 11 (35.5%), (18.6-52.3) | 13 (54.2%), (34.2-74.1) |  |  |
| **Sleep Difficulty** n (%), (95% CI) | 36 (24.8%), (17.8-31.9) | 5 (15.2%), (2.9-27.4) | 4 (13.3%), (1.2-25.5) | 7 (25.9%), (9.4-42.5) | 15 (48.4%), (30.8-66) | 5 (20.8%), (4.6-37.1) | 0.0102 |  |
| **Social activity** n (%), (95% CI) |  |  |  |  |  |  |  |  |
| Low | 26 (17.9%), (11.7-24.2) | 10 (30.3%), (14.6-46) | 8 (26.7%), (10.8-42.5) | 4 (14.8%), (1.4-28.2) | 4 (12.9%), (1.1-24.7) | 0 (0%), (NA) | 0.00373 |  |
| Medium | 64 (44.1%), (36.1-52.2) | 14 (42.4%), (25.6-59.3) | 12 (40%), (22.5-57.5) | 17 (63%), (44.7-81.2) | 14 (45.2%), (27.6-62.7) | 7 (29.2%), (11-47.4) |  |  |
| High | 55 (37.9%), (30-45.8) | 9 (27.3%), (12.1-42.5) | 10 (33.3%), (16.5-50.2) | 6 (22.2%), (6.5-37.9) | 13 (41.9%), (24.6-59.3) | 17 (70.8%), (52.6-89) |  |  |
| **Exercise,** n (%), (95% CI) | 95 (65.5%), (57.8-73.3) | 23 (69.7%), (54-85.4) | 18 (60%), (42.5-77.5) | 16 (59.3%), (40.7-77.8) | 17 (54.8%), (37.3-72.4) | 21 (87.5%), (74.3-100) | 0.0944 |  |
| Outside | 89 (61.4%), (53.5-69.3) | 23 (69.7%), (54-85.4) | 17 (56.7%), (38.9-74.4) | 14 (51.9%), (33-70.7) | 15 (48.4%), (30.8-66) | 20 (83.3%), (68.4-98.2) |  |  |
| Inside, | 6 (4.1%), (0.9-7.4) | 0 (0%), (NA) | 1 (3.3%), (0-9.8) | 2 (7.4%), (0-17.3) | 2 (6.5%), (0-15.1) | 1 (4.2%), (0-12.2) |  |  |
| **Smokers,** n (%), (95% CI) | 17 (11.7%), (6.5-17) | 0 (0%), (NA) | 7 (23.3%), (8.2-38.5) | 5 (18.5%), (3.9-33.2) | 4 (12.9%), (1.1-24.7) | 1 (4.2%), (0-12.2) | 0.0128 |  |
| **Vitamins**, n (%), (95% CI) | 62 (42.8%), (34.7-50.8) | 10 (30.3%), (14.6-46) | 14 (46.7%), (28.8-64.5) | 7 (25.9%), (9.4-42.5) | 13 (41.9%), (24.6-59.3) | 18 (75%), (57.7-92.3) | 0.0036 |  |
| **Omega-3**, n (%), (95% CI) | 22 (15.2%), (9.3-21) | 4 (12.1%), (1-23.3) | 2 (6.7%), (0-15.6) | 1 (3.7%), (0-10.8) | 4 (12.9%), (1.1-24.7) | 11 (45.8%), (25.9-65.8) | <0.001 |  |
| **Zinc**, n (%), (95% CI) | 10 (6.9%), (2.8-11) | 3 (9.1%), (0-18.9) | 6 (20%), (5.7-34.3) | 0 (0%), (NA) | 1 (3.2%), (0-9.4) | 0 (0%), (NA) | 0.0137 |  |
| **Prebiotic,** n (%), (95% CI) | 1 (0.7%), (0-2) | 1 (3%), (0-8.9) | 0 (0%), (NA) | 0 (0%), (NA) | 0 (0%), (NA) | 0 (0%), (NA) | 1 |  |
| **Probiotic,** n (%), (95% CI) | 4 (2.8%), (0.1-5.4) | 1 (3%), (0-8.9) | 1 (3.3%), (0-9.8) | 0 (0%), (NA) | 2 (6.5%), (0-15.1) | 0 (0%), (NA) | 0.776 |  |
| Marital status, n (%), (95% CI) |  |  |  |  |  |  |  |  |
| Single | 49 (33.8%), (26.1-41.5) | 28 (84.8%), (72.6-97.1) | 10 (33.3%), (16.5-50.2) | 6 (22.2%), (6.5-37.9) | 3 (9.7%), (0-20.1) | 2 (8.3%), (0-19.4) | <0.001 |  |
| Married | 96 (66.2%), (58.5-73.9) | 5 (15.2%), (2.9-27.4) | 20 (66.7%), (49.8-83.5) | 21 (77.8%), (62.1-93.5) | 28 (90.3%), (79.9-100) | 22 (91.7%), (80.6-100) |  |  |
| Lives with pet, n (%), (95% CI) | 22 (15.2%), (9.3-21) | 5 (15.2%), (2.9-27.4) | 3 (10%), (0-20.7) | 8 (29.6%), (12.4-46.9) | 4 (12.9%), (1.1-24.7) | 2 (8.3%), (0-19.4) | 0.267 |  |
| Allergy, n (%), (95% CI) | 25 (17.2%), (11.1-23.4) | 7 (21.2%), (7.3-35.2) | 6 (20%), (5.7-34.3) | 7 (25.9%), (9.4-42.5) | 3 (9.7%), (0-20.1) | 2 (8.3%), (0-19.4) | 0.337 |  |
| Thyroid_Disorder, n (%), (95% CI) | 5 (3.4%), (0.5-6.4) | 0 (0%), (NA) | 1 (3.3%), (0-9.8) | 1 (3.7%), (0-10.8) | 2 (6.5%), (0-15.1) | 1 (4.2%), (0-12.2) | 0.712 |  |
| Ulcers, n (%), (95% CI) | 0 (0%), (NA) | 0 (0%), (NA) | 0 (0%), (NA) | 0 (0%), (NA) | 0 (0%), (NA) | 0 (0%), (NA) | NA |  |
| Bites_Nails, n (%), (95% CI) | 11 (7.6%), (3.3-11.9) | 3 (9.1%), (0-18.9) | 7 (23.3%), (8.2-38.5) | 1 (3.7%), (0-10.8) | 0 (0%), (NA) | 0 (0%), (NA) | 0.00282 |  |
| Constipation, n (%), (95% CI) | 19 (13.1%), (7.6-18.6) | 3 (9.1%), (0-18.9) | 3 (10%), (0-20.7) | 3 (11.1%), (0-23) | 6 (19.4%), (5.4-33.3) | 4 (16.7%), (1.8-31.6) | 0.732 |  |
| Dyslipidemia, n (%), (95% CI) | 47 (32.4%), (24.8-40) | 9 (27.3%), (12.1-42.5) | 14 (46.7%), (28.8-64.5) | 11 (40.7%), (22.2-59.3) | 6 (19.4%), (5.4-33.3) | 7 (29.2%), (11-47.4) | 0.161 |  |
| Hospitalization rare, n (%), (95% CI) | 145 (100%), (100-100) | 33 (100%), (100-100) | 30 (100%), (100-100) | 27 (100%), (100-100) | 31 (100%), (100-100) | 24 (100%), (100-100) | 0.786 |  |
| Antihistamines, n (%), (95% CI) | 18 (12.4%), (7-17.8) | 4 (12.1%), (1-23.3) | 5 (16.7%), (3.3-30) | 5 (18.5%), (3.9-33.2) | 3 (9.7%), (0-20.1) | 1 (4.2%), (0-12.2) | 0.534 |  |
| Insulin, n (%), (95% CI) | 0 (0%), (NA) | 0 (0%), (NA) | 0 (0%), (NA) | 0 (0%), (NA) | 0 (0%), (NA) | 0 (0%), (NA) | NA |  |
| Metformin, n (%), (95% CI) | 0 (0%), (NA) | 0 (0%), (NA) | 0 (0%), (NA) | 0 (0%), (NA) | 0 (0%), (NA) | 0 (0%), (NA) | NA |  |
| Antihypertensive, n (%), (95% CI) | 0 (0%), (NA) | 0 (0%), (NA) | 0 (0%), (NA) | 0 (0%), (NA) | 0 (0%), (NA) | 0 (0%), (NA) | NA |  |
| Analgesic, n (%), (95% CI) | 80 (55.2%), (47.1-63.3) | 13 (39.4%), (22.7-56.1) | 14 (46.7%), (28.8-64.5) | 16 (59.3%), (40.7-77.8) | 22 (71%), (55-86.9) | 15 (62.5%), (43.1-81.9) | 0.0905 |  |
| Laxative, n (%), (95% CI) | 2 (1.4%), (0-3.3) | 0 (0%), (NA) | 0 (0%), (NA) | 1 (3.7%), (0-10.8) | 1 (3.2%), (0-9.4) | 0 (0%), (NA) | 0.633 |  |
| Heart_Treatment, n (%), (95% CI) | 0 (0%), (NA) | 0 (0%), (NA) | 0 (0%), (NA) | 0 (0%), (NA) | 0 (0%), (NA) | 0 (0%), (NA) | NA |  |
| Magnesium, n (%), (95% CI) | 10 (6.9%), (2.8-11) | 1 (3%), (0-8.9) | 4 (13.3%), (1.2-25.5) | 0 (0%), (NA) | 2 (6.5%), (0-15.1) | 3 (12.5%), (0-25.7) | 0.183 |  |
| Abbreviations: BMI, body mass index; SD, standard deviation; CI, confidence interval. Continuous variables are presented as mean ± SD with corresponding 95% confidence intervals (CI). Categorical variables are presented as n (%) with corresponding 95% CI. P-values were obtained using two-sided Kruskal–Wallis tests for continuous variables and two-sided Fisher’s exact tests for categorical variables. Analyses were performed in R (v4.5.1). | | | | | | | |  |
|  |  |  |  |  |  |  |  |  |
|  |  |  |  |  |  |  |  |  |

**Table S2: Pairwise PERMANOVA results for beta diversity comparisons between age groups.**

| Group A | Group B | F_model | R^2^ | P_value | Q_value |
| --- | --- | --- | --- | --- | --- |
| 1 | 2 | 1.230 | 0.228 | 0.008 | 0.080 |
| 1 | 3 | 1.209 | 0.236 | 0.019 | 0.095 |
| 1 | 4 | 1.119 | 0.208 | 0.094 | 0.313 |
| 1 | 5 | 1.078 | 0.246 | 0.163 | 0.408 |
| 2 | 3 | 1.043 | 0.221 | 0.306 | 0.507 |
| 2 | 4 | 1.045 | 0.207 | 0.252 | 0.504 |
| 2 | 5 | 0.968 | 0.239 | 0.639 | 0.799 |
| 3 | 4 | 0.784 | 0.173 | 0.999 | 0.999 |
| 3 | 5 | 1.020 | 0.244 | 0.355 | 0.507 |
| 4 | 5 | 0.861 | 0.215 | 0.985 | 0.999 |

PERMANOVA was performed on Bray–Curtis dissimilarities (adonis2; 999 permutations). Pairwise comparisons were performed across age groups (G1: 19–29 y; G2: 30–39 y; G3: 40–49 y; G4: 50–59 y; G5: 60–69 y). The table reports the pseudo-F statistic (F_model), effect size (R²), permutation p-values, and Benjamini–Hochberg FDR–adjusted q-values (correction applied across the family of pairwise tests).

**
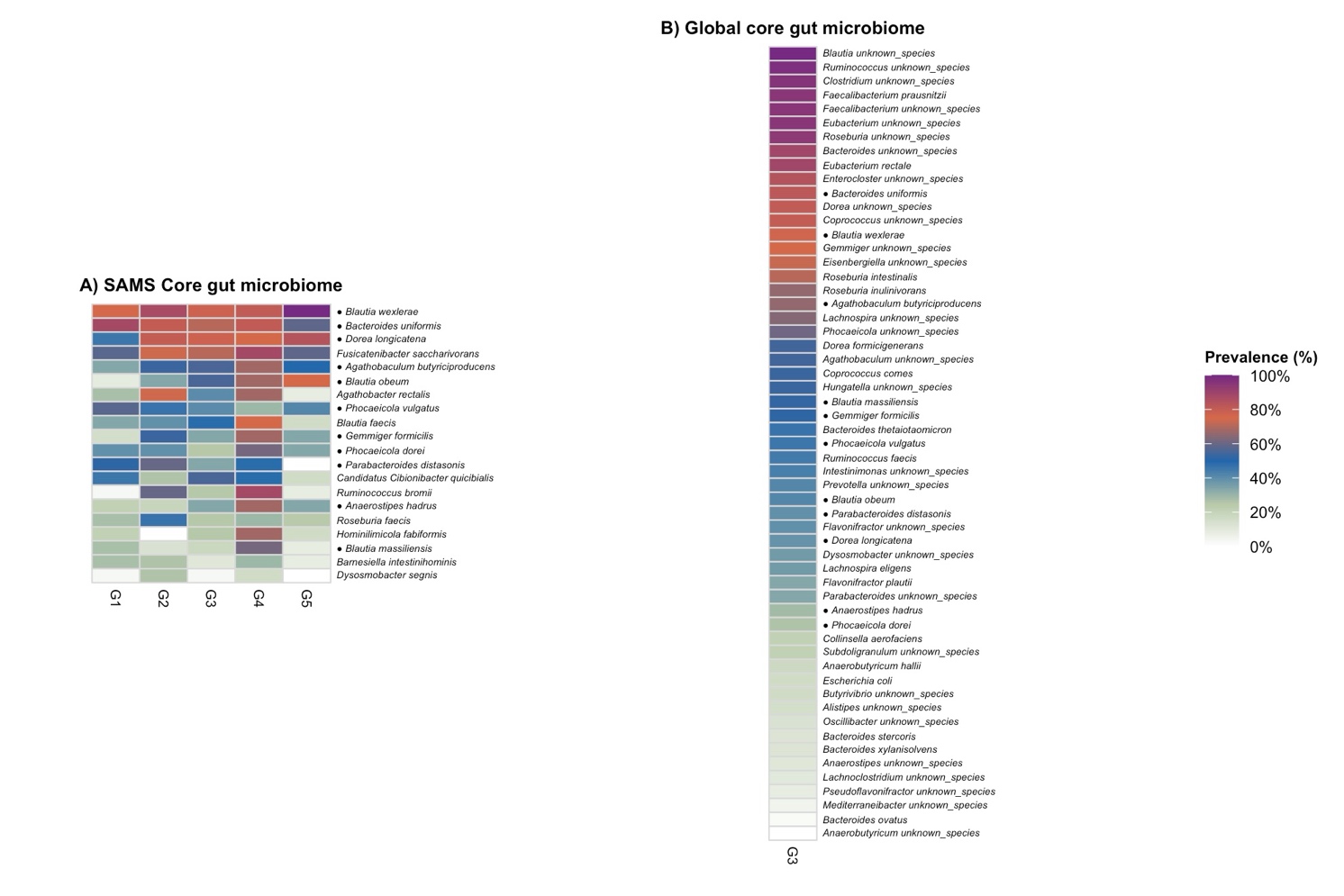
**

**Supplementary Figure 2:** Core microbiome overlaps between the SAMS cohort and a global reference cohort**.** Core taxa were defined as species present in ≥50% of samples with a mean relative abundance ≥0.1%. (A) The overall SAMS core microbiome, defined as the union of core taxa across all age groups (G1–G5). (B) An age-matched global core microbiome (Group 3; 18–64 years) derived from the dataset reported by Mancabelli et al. (2024). Species names were harmonized at the species level prior to comparison. Taxa shared between the SAMS core **(A)** and the global core **(B)** are indicated by filled circles (●), whereas non-circled taxa represent cohort-specific core species. Heatmaps display the prevalence of each core species, with color intensity corresponding to percentage prevalence as indicated by the color bar.

**Table S3: Core fecal microbiome species among healthy age groups (G1–G5) in Saudi Aging and Microbiome Study (SAMS) cohort.**

| **Species** | **Prevalence >50% and relative average abundance >0.1%** | | | | | | | | | | **Relative average abundance** | | | | | | | | | |
| --- | --- | --- | --- | --- | --- | --- | --- | --- | --- | --- | --- | --- | --- | --- | --- | --- | --- | --- | --- | --- |
|  | **G 1 19–29 n=33** | | | **G 2 30–39 n=30** | **G 3 40–49 n=27** | | | **G 4 50–59 n=31** | **G 5 60–69 n=24** | | **G 1 19–29 n=33** | **G2 30–39 n=30** | | | **G 3 40–49 n=27** | | **G 4 50–59 n=31** | | **G 5 60–69 n=24** | |
| *Agathobacter rectalis* | | 63.64% | 86.67% | | | 70.37% | 83.87% | | | 54.17% | 2.20% | | 3.44% | 2.66% | | 4.43% | | 1.35% | |  |
| *Agathobaculum butyriciproducens* | | 66.67% | 76.67% | | | 77.78% | 83.87% | | | 75.00% | 0.21% | | 0.26% | 0.28% | | 0.43% | | 0.55% | |  |
| *Anaerostipes hadrus* | | 60.61% | 60.00% | | | 66.67% | 83.87% | | | 66.67% | 0.49% | | 0.71% | 0.45% | | 0.53% | | 0.95% | |  |
| *Bacteroides uniformis* | | 93.94% | 90.00% | | | 85.19% | 90.32% | | | 79.17% | 5.06% | | 3.08% | 3.33% | | 3.06% | | 2.47% | |  |
| *Barnesiella intestinihominis* | | 63.64% | 63.33% | | | 55.56% | 64.52% | | | 54.17% | 1.06% | | 0.67% | 0.69% | | 0.60% | | 0.49% | |  |
| *Blautia faecis* | | 66.67% | 70.00% | | | 74.07% | 87.10% | | | 58.33% | 0.51% | | 0.38% | 0.59% | | 0.79% | | 0.63% | |  |
| *Blautia massiliensis* | | 63.64% | 56.67% | | | 59.26% | 80.65% | | | 54.17% | 0.80% | | 0.52% | 0.42% | | 0.71% | | 0.45% | |  |
| *Blautia obeum* | | 54.55% | 66.67% | | | 77.78% | 83.87% | | | 87.50% | 0.23% | | 0.25% | 0.68% | | 1.02% | | 1.34% | |  |
| *Blautia wexlerae* | | 87.88% | 93.33% | | | 88.89% | 90.32% | | | 100.00% | 1.55% | | 2.10% | 1.87% | | 2.02% | | 3.39% | |  |
| *Candidatus Cibionibacter quicibialis* | | 72.73% | 63.33% | | | 77.78% | 74.19% | | | 58.33% | 1.12% | | 0.66% | 0.66% | | 0.93% | | 0.37% | |  |
| *Dorea longicatena* | | 72.73% | 90.00% | | | 88.89% | 87.10% | | | 91.67% | 0.64% | | 0.71% | 0.78% | | 0.78% | | 1.32% | |  |
| *Dysosmobacter segnis* | | 51.52% | 63.33% | | | 51.85% | 58.06% | | | 50.00% | 0.43% | | 0.41% | 0.32% | | 0.28% | | 0.16% | |  |
| *Fusicatenibacter saccharivorans* | | 78.79% | 86.67% | | | 85.19% | 93.55% | | | 79.17% | 1.00% | | 1.25% | 1.13% | | 1.41% | | 2.56% | |  |
| *Gemmiger formicilis* | | 57.58% | 76.67% | | | 66.67% | 83.87% | | | 66.67% | 0.44% | | 0.59% | 0.96% | | 0.82% | | 0.94% | |  |
| *Hominilimicola fabiformis* | | 60.61% | 50.00% | | | 62.96% | 83.87% | | | 58.33% | 0.17% | | 0.28% | 0.40% | | 0.53% | | 0.50% | |  |
| *Parabacteroides distasonis* | | 75.76% | 80.00% | | | 66.67% | 74.19% | | | 50.00% | 0.95% | | 0.72% | 0.45% | | 0.82% | | 0.42% | |  |
| *Phocaeicola dorei* | | 69.70% | 70.00% | | | 62.96% | 80.65% | | | 66.67% | 2.45% | | 1.88% | 2.64% | | 2.10% | | 1.68% | |  |
| *Phocaeicola vulgatus* | | 78.79% | 73.33% | | | 70.37% | 64.52% | | | 70.83% | 7.69% | | 3.27% | 2.64% | | 1.59% | | 4.13% | |  |
| *Roseburia faecis* | | 63.64% | 73.33% | | | 62.96% | 64.52% | | | 62.50% | 1.38% | | 1.44% | 1.18% | | 1.20% | | 0.97% | |  |
| *Ruminococcus bromii* | | 51.52% | 80.00% | | | 62.96% | 93.55% | | | 54.17% | 1.71% | | 1.81% | 1.62% | | 2.55% | | 1.85% | |  |
| *Alistipes communis* | | 36.36% | 63.33% | | | 44.44% | 35.48% | | | 37.50% | 0.19% | | 0.44% | 0.21% | | 0.18% | | 0.21% | |  |
| *Blautia_sp MCC283* | | 15.15% | 40.00% | | | 25.93% | 25.81% | | | 58.33% | 0.22% | | 0.41% | 0.09% | | 0.18% | | 1.14% | |  |
| *Clostridium_sp AM22_11AC* | | 42.42% | 46.67% | | | 37.04% | 51.61% | | | 41.67% | 0.21% | | 0.40% | 0.32% | | 0.24% | | 0.65% | |  |
| *Coprococcus eutactus* | | 24.24% | 33.33% | | | 44.44% | 54.84% | | | 45.83% | 0.31% | | 0.30% | 0.99% | | 1.11% | | 0.50% | |  |
| *Lawsonibacter asaccharolyticus* | | 36.36% | 56.67% | | | 33.33% | 35.48% | | | 37.50% | 0.10% | | 0.18% | 0.13% | | 0.09% | | 0.10% | |  |
| *Oscillibacter_sp_MSJ_31* | | 30.30% | 43.33% | | | 51.85% | 41.94% | | | 33.33% | 0.19% | | 0.46% | 0.38% | | 0.16% | | 0.20% | |  |
| *Ruminococcus torques* | | 27.27% | 46.67% | | | 29.63% | 32.26% | | | 50.00% | 0.14% | | 0.40% | 0.17% | | 0.19% | | 0.30% | |  |

**Table S4: Core fecal microbiome species reprorted in global dataset (Mancabelli et al., 2024).**

|  | **Prevalence >50% and relative average abundance >0.1%** | **Relative average abundance** |
| --- | --- | --- |
| **Species** | **G3** (18–64 years)  n= 2,632 | **G3** (18–64 years)  n= 2,632 |
| *Escherichia coli* | 58.51% | 1.33% |
| *Bacteroides unknown* | 92.90% | 1.23% |
| *Clostridium unknown_species* | 96.12% | 0.60% |
| *Blautia unknown_species* | 98.52% | 2.08% |
| *Bacteroides uniformis* | 89.40% | 3.40% |
| *Ruminococcus unknown_species* | 97.26% | 1.68% |
| *Blautia wexlerae* | 87.42% | 1.68% |
| *Flavonifractor plautii* | 66.53% | 0.29% |
| *Phocaeicola vulgatus* | 72.49% | 1.15% |
| *Bacteroides thetaiotaomicron* | 72.72% | 0.59% |
| *Phocaeicola dorei* | 63.11% | 1.29% |
| *Eubacterium unknown_species* | 95.90% | 1.48% |
| *Parabacteroides distasonis* | 69.38% | 0.87% |
| *Enterocloster unknown_species* | 90.35% | 0.22% |
| *Roseburia unknown_species* | 95.59% | 1.14% |
| *Faecalibacterium unknown_species* | 96.01% | 3.23% |
| *Faecalibacterium prausnitzii* | 96.05% | 2.94% |
| *Bacteroides xylanisolvens* | 56.34% | 0.56% |
| *Phocaeicola unknown_species* | 79.71% | 0.65% |
| *Anaerostipes hadrus* | 63.87% | 1.09% |
| *Coprococcus unknown_species* | 88.87% | 0.75% |
| *Eubacterium rectale* | 92.86% | 4.11% |
| *Blautia massiliensis* | 75.68% | 0.77% |
| *Dorea unknown_species* | 89.02% | 0.58% |
| *Bacteroides ovatus* | 51.41% | 0.22% |
| *Roseburia intestinalis* | 84.38% | 1.01% |
| *Gemmiger unknown_species* | 86.06% | 0.87% |
| *Eisenbergiella unknown_species* | 85.41% | 0.18% |
| *Gemmiger formicilis* | 75.23% | 1.09% |
| *Blautia obeum* | 70.33% | 0.52% |
| *Agathobaculum butyriciproducens* | 81.76% | 0.48% |
| *Alistipes unknown_species* | 57.60% | 0.50% |
| *Intestinimonas unknown_species* | 71.43% | 0.40% |
| *Dorea formicigenerans* | 76.82% | 0.52% |
| *Ruminococcus faecis* | 71.81% | 1.15% |
| *Flavonifractor unknown_species* | 69.34% | 0.28% |
| *Parabacteroides unknown_species* | 66.45% | 0.19% |
| *Dorea longicatena* | 69.00% | 0.58% |
| *Agathobaculum unknown_species* | 76.52% | 0.35% |
| *Anaerobutyricum hallii* | 58.81% | 0.66% |
| *Dysosmobacter unknown_species* | 68.01% | 0.38% |
| *Coprococcus comes* | 76.22% | 0.54% |
| *Collinsella aerofaciens* | 60.68% | 1.31% |
| *Roseburia inulinivorans* | 82.07% | 1.20% |
| *Subdoligranulum unknown_species* | 60.60% | 0.24% |
| *Oscillibacter unknown_species* | 56.69% | 0.22% |
| *Lachnospira unknown_species* | 81.12% | 0.91% |
| *Pseudoflavonifractor unknown_species* | 54.45% | 0.16% |
| *Anaerostipes unknown_species* | 55.74% | 0.22% |
| *Prevotella unknown_species* | 70.48% | 7.09% |
| *Bacteroides stercoris* | 56.42% | 1.56% |
| *Lachnospira eligens* | 67.97% | 0.96% |
| *Anaerobutyricum unknown_species* | 50.34% | 0.19% |
| *Hungatella unknown_species* | 76.14% | 0.11% |
| *Butyrivibrio unknown_species* | 58.13% | 0.15% |
| *Lachnoclostridium unknown_species* | 55.32% | 0.12% |
| *Mediterraneibacter unknown_species* | 52.89% | 0.14% |

| **Table S5: Age-Associated Correlation Statistics for Microbial Phyla and Species** | | | | | | | |
| --- | --- | --- | --- | --- | --- | --- | --- |
| **Phylum/ Species** | **Spearman's p** | **Effective sample size** | **Number of covariates** | **95% CI (lower)** | **95% CI (upper)** | **Partial spearman p-value** | **FDR-adjusted q-value** |
| *Actinomycetota* | 0.2641 | 145 | 10 | 0.1182 | 0.4100 | 0.3362 | 0.5324 |
| *Bacteria unclassified* | 0.0323 | 145 | 10 | -0.1017 | 0.1699 | 0.2155 | 0.4549 |
| *Bacteroidota* | -0.3972 | 145 | 10 | -0.5469 | -0.2389 | 0.0001 | 0.0020 |
| *Campylobacterota* | 0.0151 | 145 | 10 | -0.1293 | 0.1767 | 0.3316 | 0.5324 |
| *Candidatus Melainabacteria* | 0.1612 | 145 | 10 | 0.0035 | 0.3279 | 0.2395 | 0.4551 |
| *Candidatus Saccharibacteria* | -0.1325 | 145 | 10 | -0.2415 | -0.1255 | 0.1214 | 0.3295 |
| *Chloroflexota* | -0.0376 | 145 | 10 | -0.1807 | 0.1101 | 0.8594 | 0.9680 |
| *Elusimicrobiota* | -0.0568 | 145 | 10 | -0.1644 | 0.0660 | 0.2123 | 0.4549 |
| *Firmicutes* | 0.3432 | 145 | 10 | 0.1800 | 0.4908 | 0.0006 | 0.0060 |
| *Fusobacteriota* | -0.0351 | 145 | 10 | -0.0971 | 0.0198 | 0.9943 | 0.9943 |
| *Lentisphaerota* | -0.0458 | 145 | 10 | -0.2035 | 0.1230 | 0.3822 | 0.5586 |
| *Methanobacteriota* | 0.0464 | 145 | 10 | -0.1190 | 0.2133 | 0.4635 | 0.6291 |
| *Mycoplasmatota* | -0.0176 | 145 | 10 | -0.1677 | 0.1265 | 0.5308 | 0.6724 |
| *Pseudomonadota* | 0.1680 | 145 | 10 | 0.0022 | 0.3271 | 0.0375 | 0.1424 |
| *Spirochaetota* | 0.1036 | 145 | 10 | 0.0906 | 0.1999 | 0.0750 | 0.2376 |
| *Synergistota* | 0.0922 | 145 | 10 | -0.0493 | 0.2066 | 0.0264 | 0.1256 |
| *Thermodesulfobacteriota* | -0.1418 | 145 | 10 | -0.2956 | 0.0135 | 0.9682 | 0.9943 |
| *Thermoplasmatota* | 0.1042 | 145 | 10 | 0.0348 | 0.1884 | 0.0254 | 0.1256 |
| *Verrucomicrobiota* | 0.0538 | 145 | 10 | -0.1112 | 0.2206 | 0.8661 | 0.9680 |
| *Agathobacter rectalis* | -0.0247 | 145 | 10 | -0.1994 | 0.1385 | 0.0380 | 0.1627 |
| *Akkermansia muciniphila* | 0.0578 | 145 | 10 | -0.0940 | 0.2228 | 0.6508 | 0.7740 |
| *Alistipes onderdonkii* | -0.1763 | 145 | 10 | -0.3459 | 0.0029 | 0.2989 | 0.5606 |
| *Alistipes putredinis* | -0.1960 | 145 | 10 | -0.3479 | -0.0223 | 0.3839 | 0.6775 |
| *Bacteroides caccae* | -0.1980 | 145 | 10 | -0.3569 | -0.0221 | 0.0092 | 0.0692 |
| *Bacteroides ovatus* | -0.1361 | 145 | 10 | -0.2940 | 0.0387 | 0.0283 | 0.1417 |
| *Bacteroides stercoris* | 0.0879 | 145 | 10 | -0.0711 | 0.2456 | 0.1953 | 0.4507 |
| *Bacteroides thetaiotaomicron* | -0.3499 | 145 | 10 | -0.4989 | -0.1893 | 0.0026 | 0.0260 |
| *Bacteroides uniformis* | -0.2569 | 145 | 10 | -0.4005 | -0.0931 | 0.0937 | 0.2810 |
| *Barnesiella intestinihominis* | -0.1966 | 145 | 10 | -0.3531 | -0.0309 | 0.0802 | 0.2673 |
| *Bifidobacterium adolescentis* | 0.1789 | 145 | 10 | 0.0173 | 0.3256 | 0.2990 | 0.5606 |
| *Blautia obeum* | 0.4575 | 145 | 10 | 0.3114 | 0.5792 | 0.0009 | 0.0260 |
| *Blautia wexlerae* | 0.1870 | 145 | 10 | 0.0255 | 0.3403 | 0.2405 | 0.5154 |
| *Candidatus Cibionibacter quicibialis* | -0.0228 | 145 | 10 | -0.1883 | 0.1430 | 0.6814 | 0.7740 |
| *Dorea longicatena* | 0.2502 | 145 | 10 | 0.0898 | 0.4092 | 0.6032 | 0.7740 |
| *Faecalibacterium prausnitzii* | -0.1714 | 145 | 10 | -0.3202 | -0.0078 | 0.1474 | 0.3925 |
| *Fusicatenibacter saccharivorans* | 0.1567 | 145 | 10 | -0.0033 | 0.3138 | 0.6906 | 0.7740 |
| *Gemmiger formicilis* | 0.1406 | 145 | 10 | -0.0142 | 0.2920 | 0.0157 | 0.0939 |
| *Leyella stercorea* | -0.0219 | 145 | 10 | -0.1749 | 0.1222 | 0.7224 | 0.7740 |
| *Parabacteroides distasonis* | -0.2469 | 145 | 10 | -0.4037 | -0.0877 | 0.1570 | 0.3925 |
| *Phocaeicola dorei* | 0.0252 | 145 | 10 | -0.1368 | 0.1958 | 0.4419 | 0.6978 |
| *Phocaeicola vulgatus* | -0.1704 | 145 | 10 | -0.3296 | 0.0111 | 0.0018 | 0.0260 |
| *Roseburia faecis* | -0.0949 | 145 | 10 | -0.2504 | 0.0575 | 0.4311 | 0.6978 |
| *Ruminococcus bicirculans* | -0.0678 | 145 | 10 | -0.2198 | 0.1041 | 0.5935 | 0.7740 |
| *Ruminococcus bromii* | 0.1160 | 145 | 10 | -0.0639 | 0.2939 | 0.0664 | 0.2490 |
| *Segatella brasiliensis* | 0.1185 | 145 | 10 | 0.0016 | 0.2314 | 0.7965 | 0.8240 |
| *Segatella brunsvicensis* | -0.0296 | 145 | 10 | -0.1841 | 0.1537 | 0.7148 | 0.7740 |
| *Segatella copri* | 0.0483 | 145 | 10 | -0.1007 | 0.2073 | 0.9452 | 0.9452 |
| *Segatella hominis* | 0.0850 | 145 | 10 | -0.0729 | 0.2260 | 0.7209 | 0.7740 |
| *Segatella sinensis* | -0.0812 | 145 | 10 | -0.2314 | 0.0670 | 0.4713 | 0.7070 |

**Reference**

Mancabelli, L., Milani, C., Biase, R. D., Bocchio, F., Fontana, F., Gabriele, A. L., Alessandri, G., Tarracchini, C., Viappiani, A., Conto, F. D., Nouvenne, A., Ticinesi, A., Bussolati, O., Meschi, T., Cecchi, R., Turroni, F., & Ventura, M. (2024). Taxonomic and metabolic development of the human gut microbiome across life stages: A worldwide metagenomic investigation. *mSystems*, *9*(4), e01294-23. https://doi.org/10.1128/msystems.01294-23
